# Water drinking during aerobic exercise improves the recovery of non-linear heart rate dynamics in coronary artery disease: crossover clinical trial

**DOI:** 10.3389/fnins.2023.1147299

**Published:** 2023-06-22

**Authors:** Maria Júlia Lopez Laurino, Anne Kastelianne França da Silva, Lorena Altafin Santos, Luiz Carlos Marques Vanderlei

**Affiliations:** Laboratory of Stress Physiology, Department of Physiotherapy, São Paulo State University (UNESP), Faculty of Science and Technology, Presidente Prudente, Brazil

**Keywords:** autonomic nervous system, cardiac rehabilitation, complexity, fluid replacement, post-exercise recovery, coronary artery disease

## Abstract

**Introduction:**

The post-exercise recovery is a period of vulnerability of the cardiovascular system in which autonomic nervous system plays a key role in cardiovascular deceleration. It is already known that individuals with coronary artery disease (CAD) are at greater risk due to delayed vagal reactivation in this period. Water ingestion has been studied as a strategy to improve autonomic recovery and mitigate the risks during recovery. However, the results are preliminary and need further confirmation. Therefore, our aim was to investigate the influence of individualized water drinking on the non-linear dynamics of heart rate during and after aerobic exercise in CAD subjects.

**Methods:**

30 males with CAD were submitted to a control protocol composed of initial rest, warming up, treadmill exercise, and passive recovery (60 min). After 48 hours they performed the hydration protocol, composed of the same activities, however, with individualized water drinking proportional to the body mass lost in the control protocol. The non-linear dynamics of heart rate were assessed by indices of heart rate variability extracted from the recurrence plot, detrended fluctuation analysis, and symbolic analysis.

**Results and discussion:**

During exercise, the responses were physiological and similar in both protocols, indicating high sympathetic activity and reduced complexity. During recovery, the responses were also physiological, indicating the rise of parasympathetic activity and the return to a more complex state. However, during hydration protocol, the return to a more complex physiologic state occurred sooner and non-linear HRV indices returned to resting values between the 5th and 20th minutes of recovery. In contrast, during the control protocol, only a few indices returned to resting values within 60 minutes. Despite that, differences between protocols were not found. We conclude that the water drinking strategy accelerated the recovery of non-linear dynamics of heart rate in CAD subjects but did not influence responses during exercise. This is the first study to characterize the non-linear responses during and after exercise in CAD subjects.

## 1. Introduction

Coronary artery disease (CAD) is associated with autonomic imbalance (Floras and Ponikowski, 2015), which delays the post-exercise recovery of the cardiovascular and autonomic nervous systems (ANS) (Ushijima et al., 2009). The recovery efficiency of these systems has a significant prognostic value (Qiu et al., 2017) and is related to the risk of occurrence of acute cardiovascular events after exercises, such as ischemia and arrhythmias (Thompson et al., 2007).

Among the strategies that can accelerate post-exercise recovery, a fluid replacement strategy can be easily implemented in exercise-based cardiac rehabilitation programs. However, despite the important role that the autonomic nervous system (ANS) plays in cardiovascular deceleration after exercise (Fisher et al., 2015) and the risks related to the autonomic impairment and delayed recovery of CAD subjects (Ushijima et al., 2009; Floras and Ponikowski, 2015), only two studies investigated the effects of water drinking in the autonomic recovery in this population (Laurino et al., 2021; Silva et al., 2022).

Results from these studies were based on heart rate variability (HRV) analysis that evaluated the autonomic recovery. HRV analysis evaluates the ANS through the variation of consecutive NN intervals. HRV can be analyzed by linear methods, such as the time and frequency domains (Shaffer and Ginsberg, 2017), and by non-linear methods, which give information regarding the level of complexity of the ANS, which reflects the parasympathetic and sympathetic interaction/competition to modulate the HR (Henriques et al., 2020).

Therefore, using the HRV indices extracted from the Poincaré plot, Silva et al. (2022) evaluated the effects of water drinking in the 1-h autonomic recovery after a 40-min moderate-intensity session of exercise and found that water drinking promoted a faster and more efficient parasympathetic recovery. Corroborating with this finding, Laurino et al. (2021) reported a small effect of water drinking in the fast recovery phase from the same exercise session, recording a higher vagal response at the first minute of recovery.

Although both studies had positive results, they considered only linear indices of HRV, and the effect found for water drinking was mild to moderate. Thus, a new investigation is needed to determine whether these results can be reproduced using other HRV metrics, which will add further evidence to the benefits of water intake in exercise recovery. In this scenario, the use of non-linear metrics appears reasonable, because ANS control over the HR works as a non-linear and complex biological system (Porta et al., 2009). Also, Figueiredo et al. (2018) have suggested that the non-linear methods are more sensitive for detecting the physiological acute modifications following a high-intensity training session.

Nevertheless, studies that evaluated autonomic modulation during and after exercise through non-linear methods are just beginning, and most have considered only one method of non-linear analysis and evaluated only healthy young men and athletes (Henriques et al., 2020).

Thus, this study aimed to investigate the influence of a programmed water drinking protocol in CAD subjects, performed during and after moderate aerobic exercise, on the non-linear dynamics of heart rate during the periods of exercise and recovery from a cardiac rehabilitation session. We hypothesized that water drinking would be able to accelerate autonomic recovery and reduce the responses of non-linear dynamics of HR during exercise.

## 2. Materials and methods

### 2.1. Trial design

The trial was a crossover design. However, randomization was not possible once the hydration strategy adopted was individualized and based on the amount of fluid lost in the control session. Although the carryover effect was not expected in this study, a 48-h washout period was adopted. All procedures were prospectively registered at ClinicalTrials.gov (NCT03198806) and described in detail by Silva et al. (2020). We followed the Consolidated Standards of Reporting Clinical Trials extension to randomized crossover trials (Dwan et al., 2019).

### 2.2. Participants

This study recruited 38 men with the main diagnosis of CAD and left ventricle ejection fraction of above 50%, who did or did not have acute myocardial infarction and belong to functional classes I and II according to the New York Heart Association (Harvey et al., 1994). The volunteers were recruited as per convenience in two cardiac rehabilitation centers located in Presidente Prudente—São Paulo, Brazil, through a previous analysis of their medical records regarding the inclusion and exclusion criteria by an independent researcher. The eligible subjects were invited to participate in the study.

Individuals who were participating in an exercise-based cardiac rehabilitation for at least 3 months without contraindications to exercise (unstable angina, significant valvular disease, respiratory diseases, non-controlled hypertension and diabetes mellitus, renal and hepatic disorders, myocarditis, electrolyte disorders, abnormal hemodynamic responses during the cardiopulmonary exercise test, and neurological and/or orthopedics disorders that could preclude the protocol performance) were included in the study. To ensure the reliability of the HRV data, individuals who were smokers, classified with “low-risk drinking or abstinence” by the Alcohol Use Disorders Identification Test (AUDIT), Lima et al. (2005) were not included (Catai et al., 2019). Individuals who did not participate in all phases of the study protocol and those who presented errors >5% in the HRV data were excluded from the final analysis (Catai et al., 2019).

The study was approved by the Committee for Ethics and Research of the Faculty of Science and Technology—UNESP (CAAE: 54864716.8.0000.5402) and followed the Declaration of Helsinki. All volunteers signed the consent form after agreeing to participate in the study.

### 2.3. Experimental procedure

**Figure 1:**
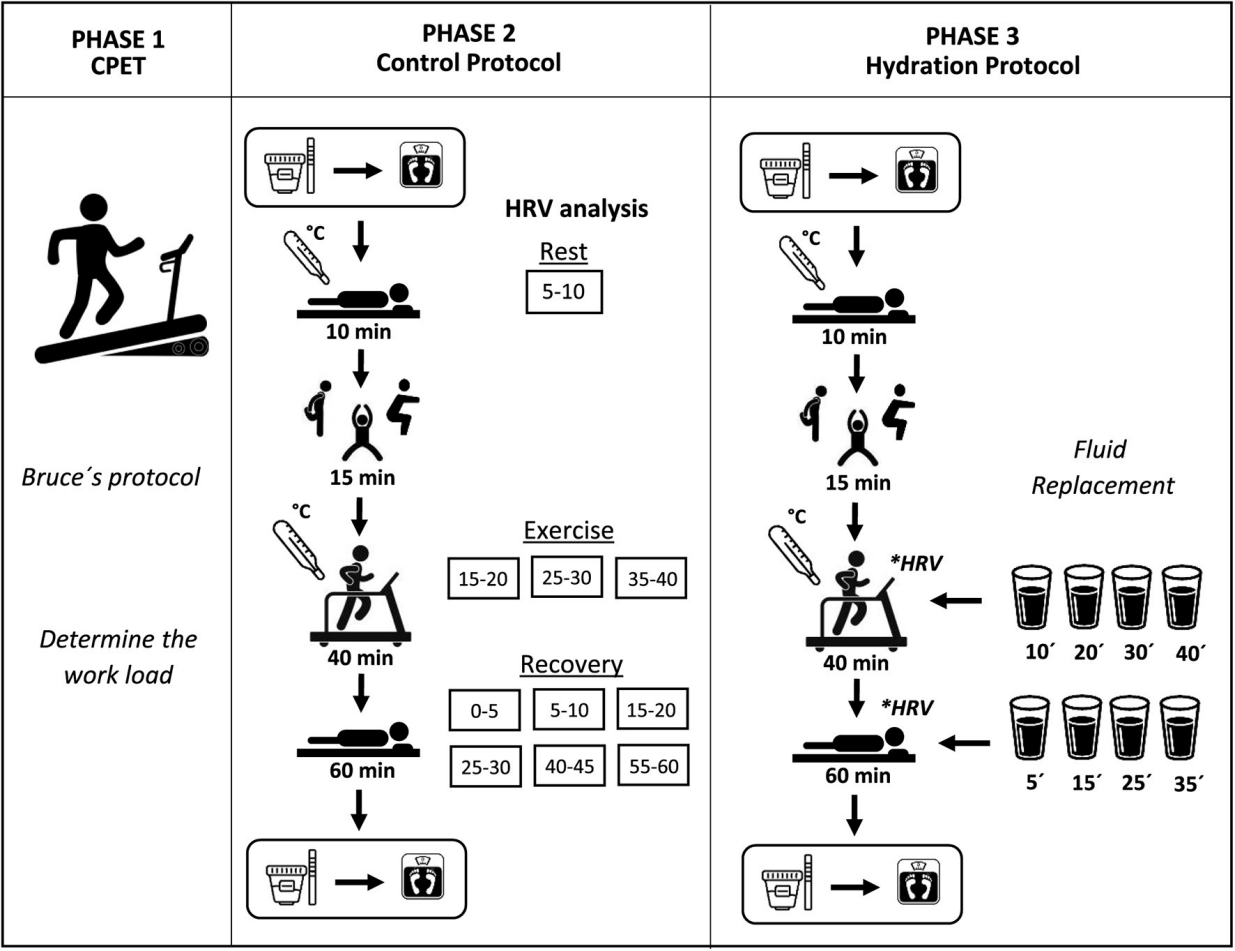
Experimental procedure.

The experimental procedure (Figure 1) was divided into three phases scheduled with an interval of at least 48 h (washout) to allow the complete recovery of the participants and minimize the acute effects of the exercise. All procedures are described in detail in the article published by Silva et al. (2020).

In the first stage, a cardiopulmonary exercise test (CPET) was performed by a physician, according to Bruce's protocol (Meneghelo et al., 2011), to evaluate the hemodynamic responses and guide the exercise intensity prescription. It was set as 60 to 80% of the HR reached at the anaerobic threshold, determined by the analysis of the expiration gases, analyzed by the commercial system Quark PFT (Cosmed, Rome, Italy) (Cabral-Santos et al., 2015). The test was interrupted by voluntary physical exertion or according to the interruption criteria for the CPET (Meneghelo et al., 2011).

In the second stage, the control protocol (CON) was performed. It was composed of activities similar to those that usually constitute a session in exercise-based cardiac rehabilitation programs: initial rest in the supine position for 10 min; warm-up (stretching and free active exercises for arms and legs) for 15 min; aerobic exercise on a treadmill for 40 min; cooldown performed for 5 min with half the load used during exercise; and passive recovery in the supine position for 60 min.

In the third stage, the hydration protocol (HYD) was performed. It was composed of the same activities and procedures as the CON except that, during exercise and recovery, all volunteers drank 8 equal portions of mineral water (Bonafont, Brazil) at room temperature, which were offered every 10 min, starting at the 10th min of the exercise (Sawka et al., 2007). The amount of water ingested by each volunteer was calculated based on the body mass variation during the CON, considering that 1 gram lost is equal to 1 milliliter of fluid lost (Von Duvillard et al., 2004). This method had the aim of matching the fluid losses during the whole protocol and avoiding hypohydration. During recovery, participants used a straw to drink the water maintaining the supine position to avoid any interference in the HRV data.

In both protocols, before the initial rest and after the passive recovery, a urine sample was collected and analyzed (Combur 10 M teste, Roche^®^, Brazil) to determine the urine-specific gravity, which is considered a marker of the hydration level (Armstrong, 2005). Values above 1.020 classified the volunteer as dehydrated (Sawka et al., 2007). After the urine collection, the body mass was measured with a precision of 5 g (Balmak, Premium Bk−200Fa, Brazil). At the end of the initial rest and cooldown, the axillar temperature was measured through a thermometer (G-Tech). To record the RR intervals used to calculate the HRV indices, the Polar RS800CX HR monitor (Polar Electro, Kempele, Finland) (Rezende Barbosa et al., 2016) was placed on the volunteer's chest.

To ensure the HRV data reliability, all volunteers were oriented to not perform vigorous physical activity and to not consume stimulant substances during the 24 h previous to each phase (Catai et al., 2019). To ensure an initial hydrated state, they were oriented to ingest 500 ml of water 2 h before CON and HYD (Sawka et al., 2007). To avoid circadian variation, all phases were performed between 1:00 and 6:00 pm, and the room temperature and humidity were controlled (21°-23°C and 40–60%) (Catai et al., 2019).

### 2.4. Outcomes

The ANS was evaluated through the HRV non-linear methods considering the following epochs for analysis: 5–10 min of initial rest; 15–20, 25–30, and 35–40 min of exercise; 0–5, 5–10, 15–20, 25–30, 40–45, and 55–60 min of recovery (Peçanha et al., 2017). We ensured that each RR series contained at least 256 consecutive RR intervals (Catai et al., 2019).

The RR series were submitted to a digital filtration (Polar Precision Performance SW version 5.0) (Nunan et al., 2009) followed by a visual inspection to eliminate premature ectopic beats and artifacts. Only series with more than 95% of sinus heartbeats were included in the study (Catai et al., 2019). The data filtering was performed by an experienced and blinded researcher.

The HRV methods considered for the ANS evaluation were as follows:

I) Detrended fluctuation analysis (DFA) is defined as the root-mean-square fluctuation of the integrated and detrended RR series. The DFA-total, the short-term alpha correlations (alpha-1), considering correlations from 4 to 11 RR intervals, and the long-term alpha correlations (alpha-2), considering the rest of the series (11 to 64 RR intervals), were calculated. This analysis classifies the RR series as linear, fractal, or random, with values ranging from 0.5 to 1.5. A resting physiological state is characterized by fractal correlation, with values closer to 1 (Peng et al., 1994). During moderate-intensity exercise, the tendency is for the RR series to become linear, with values closer to 1.5 (Gronwald et al., 2018).
II) Recurrence plot corresponds to the graph reconstruction of the spatial trajectory of the RR series (Eckmann et al., 1987). From the graph, the following indices were extracted: recurrence rate (REC—defined as the ratio of all recurrence states to all possible states), determinism (DET—defined as the ratio of recurrence points forming diagonal to all recurrence points), both of which indicate how predictable the RR series is. An RR series with higher values of REC and DET is associated with reduced parasympathetic activity, as demonstrated in animal models (Dabiré et al., 1998). For these analyses, the embedding dimension was set as *m* = 10, lag was fixed at τ = 1, and the threshold distance was determined by $r\;=\;\sqrt{m}SD$, where *SD* represents the standard deviation of the RR intervals in the time series.
III) Sample entropy (SampEn) is defined as the conditional probability that two sequences similar for n points remain similar at the next point, which indicates the RR series complexity, which is reduced during exercise (Javorka et al., 2002). For this calculation, the embedding dimension was set as *m* = 2, and the tolerance was set as *r* = 0.2*SD* (Richman and Moorman, 2000).
IV) Symbolic analysis indicates the quantity and type of variation between three consecutive RR intervals, classifying them into the following families: 0V (no variation, representing sympathetic modulation), 1V (one variation, representing global modulation), 2LV (two equal variations), and 2LV (two different variations), both representing parasympathetic modulation (Porta et al., 2007).

The DFA indices were calculated using the software “Detrended fluctuation analysis” available in the PhysioNet repository (Goldberger et al., 2000). The recurrence plot indices were calculated using the Kubios HRV software version 2.0 (Tarvainen et al., 2014). The symbolic analysis was performed using the software Symbolic Analysis fast version 4.0 (University of Milano, Italy).

As a secondary outcome, systolic (SBP) and diastolic (DBP) blood pressures were measured to provide additional information about the effect of water drinking on the cardiovascular and autonomic responses throughout the experimental procedure. The arterial blood pressure was measured indirectly using a stethoscope (Littmann, Saint Paul, USA) and an aneroid sphygmomanometer (Welch Allyn Tycos, New York, USA) at the following times: 10th min of initial rest; 15th and 35th min of treadmill exercise; and 5th, 10th, 20th, 30th, 40th, 50th, and 60th min of recovery. From these data, the mean arterial blood pressure (MBP) was calculated.

### 2.5. Sample size

The sample size was defined based on the results obtained in a pilot project for the sample entropy data. We adopted the magnitude of the significant difference of 0.2575 and considered a standard deviation of 0.3545, a significance level of 5%, and a power of 80%. Thus, the sample size resulted in 30 subjects.

### 2.6. Statistical analysis

The normality of the data was evaluated by the Shapiro–Wilk test. The comparison between the initial and final measures of body mass, axillar temperature, and urine-specific gravity was performed by Student's *t*-test for paired data or the Wilcoxon test. For the between-protocol comparison, the Student's *t*-test for unpaired data or the Mann–Whitney test was used. Cohen's *d* effect size was calculated (Maher et al., 2013). The HRV indices and arterial blood pressure were analyzed through the two-way ANOVA for repeated measures followed by Bonferroni or Dunnett's post-hoc test. The sphericity was checked by Mauchly's test, and, when violated, the Greenhouse–Geisser correction was considered. The partial eta-squared effect size was calculated (Maher et al., 2013).

This model was not adjusted by the daily use of beta-blockers and antihypertensive drugs due to the crossover design; thus, the effect of the medication is expected to not interfere with the analysis.

Statistical significance was set at 5%. The analyses were performed by a blinded researcher using a coded datasheet. All analyses were performed using the IBM SPSS Statistics software version 22.0 (IBM Corp, Armonk, New York).

## 3. Results

**Figure 2:**
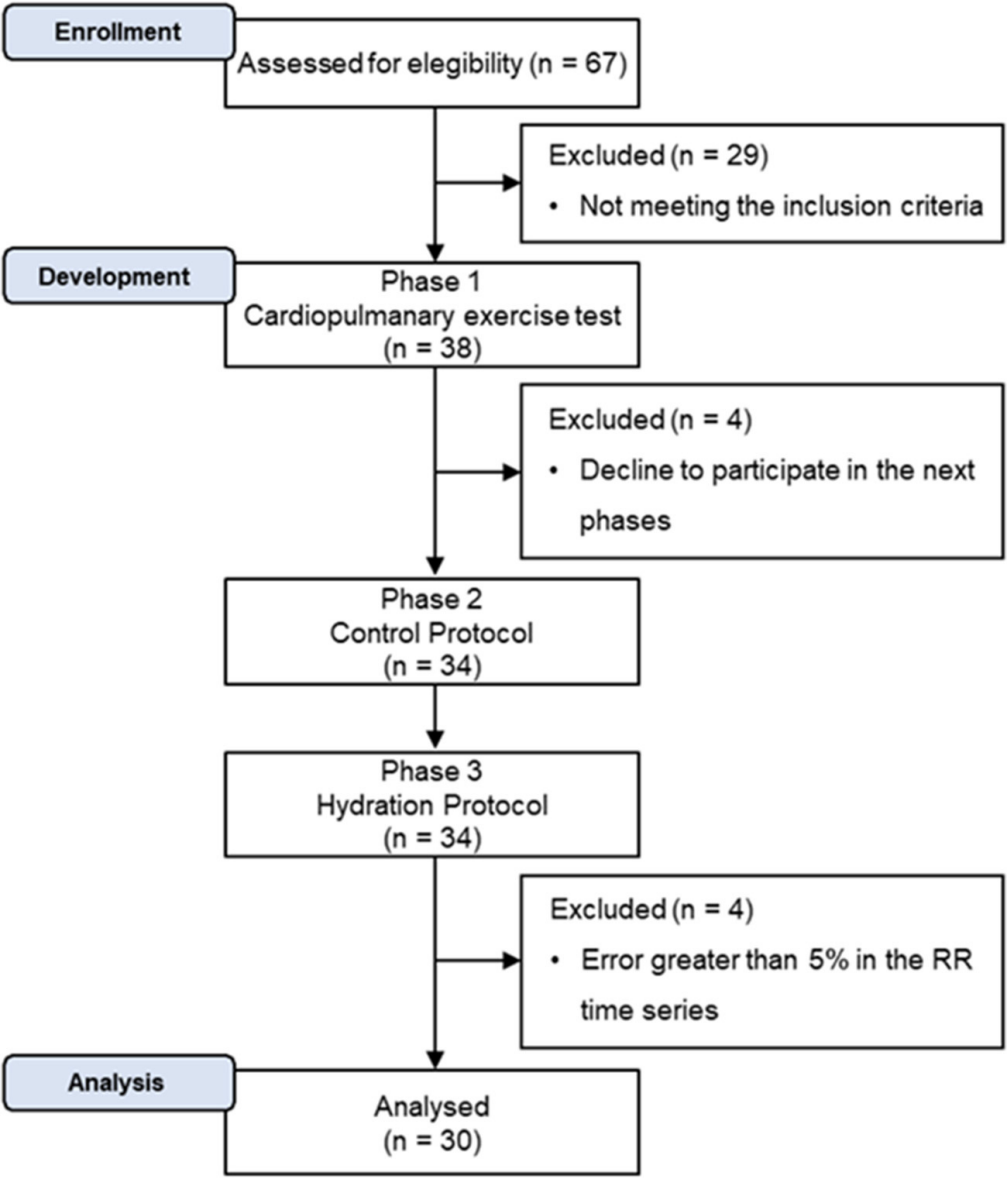
Participation flow diagram.

In this study, we initially recruited 38 men, but four subjects dropped out before CON. Thus, the outcome assessment was not possible, making it unfeasible to consider the analysis of the intention to treat. After HYD, four subjects were excluded due to equipment error at the RR interval time series record. However, the data imputation was not considered due to the repeated measures design and the great HRV variation between subjects and epochs. After sample losses, 30 subjects were analyzed (Figure 2), whose characterization is shown in Table 1.

**Table 1:** Sample characteristics (*N* = 30).

**Variable**	**Mean ±SD**	**Minimum**	**Maximum**
Age (years)	63.7 ± 8.4	45.0	83.0
Weight (Kg)	80.9 ± 12.8	58.0	108.5
Height (m)	1.7 ± 0.1	1.6	1.8
BMI (Kg/m^2^)	27.7 ± 4.1	20.6	39.4
Period of treatment (years)	3.4 ± 4.2	0.2	17.8
Resting HR (bpm)	63.3 ± 8.2	49.0	78.0
SBP (mmHg)	116.3 ± 8.9	100.0	140.0
DBP (mmHg)	76.0 ± 7.7	60.0	90.0
VO_2_peak (ml/Kg/min)	27.1 ± 6.6	14.3	38.8
Maximum HR (bpm)	138.5 ± 22.4	98.0	173.0
Treadmill workload (Km/h)	4.8 ± 0.8	3.2	6.0
Amount of water ingested (ml)	600.0 ± 180.0	200.0	1000.0
**Cardiac risk factors**	**N (%)**		
Previous myocardial infarction	4 (13.3)		
Diabetes	14 (46.7)		
Dyslipidemia	21 (70.0)		
Hypertension	26 (86.7)		
Obesity	7 (23.3)		
Family history	20 (66.7)		
Ex-smoker	13 (43.3)		
**Medicines of daily use**	**N (%)**		
Anticoagulant	23 (76.7)		
Beta-blockers	21 (70.0)		
ARA II	10 (33.3)		
Ca^2+^ channel blockers	8 (26.7)		
K^+^ channel blockers	1 (3.3)		
ACE inhibitor	6 (20.0)		
Diuretics	9 (30.0)		
Vasodilator	3 (10.0)		
Statins	24 (80.0)		
Other	11 (36.7)		

Kg, kilogram; m, meters; Kg/m^2^, kilogram/meter^2^; mmHg, millimeters of mercury; bpm, beats per minute; ml/min/kg, milliliter/minute/kilogram; km/h, kilometer/hour; BMI, body mass index; SBP, systolic blood pressure; DBP, diastolic blood pressure; HR, heart rate; VO2peak, peak oxygen consumption; ARA II, angiotensin II receptor antagonists; ACE, angiotensin-converting enzyme; Ca+, Calcium; K+ = Potassium.

Of the 30 men analyzed, seven (23.30%) did not reach the anaerobic threshold during the test, so the VO2peak value of these subjects (24.64 ± 8.28 ml/Kg/min) was considered for the exercise prescription (American College of Sports Medicine., 2014).

The fluid loss was assessed through participants' body mass measurements. Our results revealed body mass reduction after the CON protocol. The average fluid loss (600 ± 180 ml) represented a reduction of 0.7% in body mass. A small reduction in body mass was also observed after the HYD protocol. The average fluid loss (170 ± 188 ml) represented a 0.2% reduction in body mass (Table 2).

**Table 2:** Values of body mass, axillary temperature, and urine-specific gravity before and after each protocol (*N* = 30).

	**Initial**	**Final**	***P*-value (Cohen's d–effect magnitude)**
**Body mass (Kg)**			
Control	81.5 ± 12.6	**80.9** **±12.6**	< 0.001 (0.05–null*)*
Hydration	81.4 ± 12.5	**81.3** **±12.6**	0.026 (0.01–null)
**Axillary temperature (**°**C)**			
Control	35.1 ± 0.8	35.1 ± 1.0	0.328 (0.15–small)
Hydration	35.3 ± 1.0	**34.8** **±1.1**	0.042 (0.43–small)
**Urine-specific gravity**			
Control	1.015 ± 0.005	1.014 ± 0.005	0.135 (0.20–small)
Hydration	1.016 ± 0.004	**1.012** **±0.004**	< 0.001 (1.00–large)

Mean ± standard deviation. Values in bold: significant difference (p < 0.05) from the initial measure. Kg, kilogram; **°C**, degrees Celsius.

The dynamic hydration condition was assessed through urine-specific gravity. Our results revealed that the hydration level was maintained after the CON protocol. A significantly lower hydration level was observed after the HYD protocol, with a large size effect. Although no significant differences were observed, there was a reduction in the axillary temperature after the HYD protocol. No change in the axillary temperature was observed after the CON protocol (Table 2).

The non-linear HRV indices analyzed during exercise are shown in Table 3. There were no significant differences between protocols and for the time vs. protocol interaction. However, there were differences between epochs (*P*-value < 0.05) for all indices, except for SampEn, Alpha-1, and 2ULV.

**Table 3:** Comparison between rest and exercise epochs for control and hydration protocols (*N* = 30).

	**Rest**	**Exercise Epochs**			**ANOVA *P*-value (η^2^_P_-effect magnitude)**
	**5**°**-10**°**min**	**15**°**-20**°**min**	**25**°**-30**°**min**	**35**°**-40**°**min**	
**REC (%)**					
Control	34.5 ± 8.9	41.5 ± 7.0	**42.9** **±7.8**	**43.5** **±6.8**	Epochs: 0.00 (0.28–large) Interaction: 0.55 (0.01–small)
Hydration	36.0 ± 9.5	**44.5** **±8.0**	**44.3** **±8.4**	**43.1** **±5.1**		
**DET (%)**					
Control	98.0 ± 1.7	98.9 ± 1.1	99.0 ± 1.1	99.2 ± 0.9	Epochs: 0.00 (0.25–large) Interaction: 0.78 (0.00–null)
Hydration	98.2 ± 1.4	**99.2** **±0.9**	**99.1** **±1.0**	**99.3** **±0.5**		
**SampEn**					
Control	1.5 ± 0.4	1.4 ± 0.3	1.4 ± 0.3	**1.3** **±0.3**	Epochs: 0.01 (0.07–medium) Interaction: 0.79 (0.00–null)
Hydration	1.4 ± 0.4	1.4 ± 0.3	1.4 ± 0.3	1.3 ± 0.2		
**DFA-total**					
Control	1.01 ± 0.11	**1.11** **±0.14**	**1.12** **±0.13**	**1.15** **±0.16**	Epochs: 0.00 (0.18–large) Interaction: 0.95 (0.00–null)
Hydration	1.05 ± 0.16	**1.14** **±0.11**	**1.14** **±0.15**	**1.14** **±0.12**		
**Alpha-1**					
Control	0.98 ± 0.31	0.96 ± 0.25	1.01 ± 0.23	1.01 ± 0.23	Epochs: 0.54 (0.01–small) Interaction: 0.69 (0.00–null)
Hydration	1.04 ± 0.25	1.02 ± 0.21	1.02 ± 0.20	1.05 ± 0.20		
**Alpha-2**					
Control	1.01 ± 0.12	**1.10** **±0.18**	**1.10** **±0.15**	**1.10** **±0.20**	Epochs: 0.00 (0.15–large) Interaction: 0.94 (0.00–null)
Hydration	1.01 ± 0.20	**1.14** **±0.15**	**1.12** **±0.20**	**1.12** **±0.15**		
**0V (%)**					
Control	26.1 ± 16.3	32.9 ± 14.3	**34.4** **±13.7**	**38.9** **±14.6**	Epochs: 0.00 (0.14–large) Interaction: 0.24 (0.02–small)
Hydration	27.1 ± 17.5	**36.6** **±13.1**	**37.2** **±13.8**	34.9 ± 11.8		
**1V (%)**					
Control	44.1 ± 5.9	42.2 ± 6.9	42.5 ± 6.6	40.0 ± 6.9	Epochs: 0.00 (0.08–medium) Interaction: 0.32 (0.02–small)
Hydration	44.3 ± 6.8	41.1 ± 6.7	41.5 ± 6.8	41.4 ± 4.8		
**2LV (%)**					
Control	7.6 ± 4.1	**3.9** **±3.0**	**3.5** **±3.0**	**3.2** **±2.9**	Epochs: 0.00 (0.36–large) Interaction: 0.47 (0.01–small)
Hydration	7.6 ± 5.2	**2.8** **±3.6**	**2.9** **±2.3**	**3.4** **±2.4**		
**2ULV (%)**					
Control	22.2 ± 14.8	21.0 ± 8.6	19.6 ± 6.9	17.9 ± 6.9	Epochs: 0.27 (0.02–small) Interaction: 0.39 (0.01–small)
Hydration	21.0 ± 12.8	19.4 ± 10.1	18.9 ± 7.6	20.2 ± 7.5		

Mean ± standard deviation. Values in bold: significant difference (p < 0.05) from rest. REC, recurrence rate; DET, determinism; SampEn, sample entropy; DFA, analysis of detrended fluctuations; 0V, no variation; 1V, one variation; 2LV, two equal variations; 2ULV, two different variations; η^2^_P_, partial eta-squared.

During exercise, in both protocols, the indices REC, DFA-total, and Alpha-2 increased and the 2LV index decreased. The 0V index increased only in CON, and the DET index increased only during HYD.

**Figure 3:**
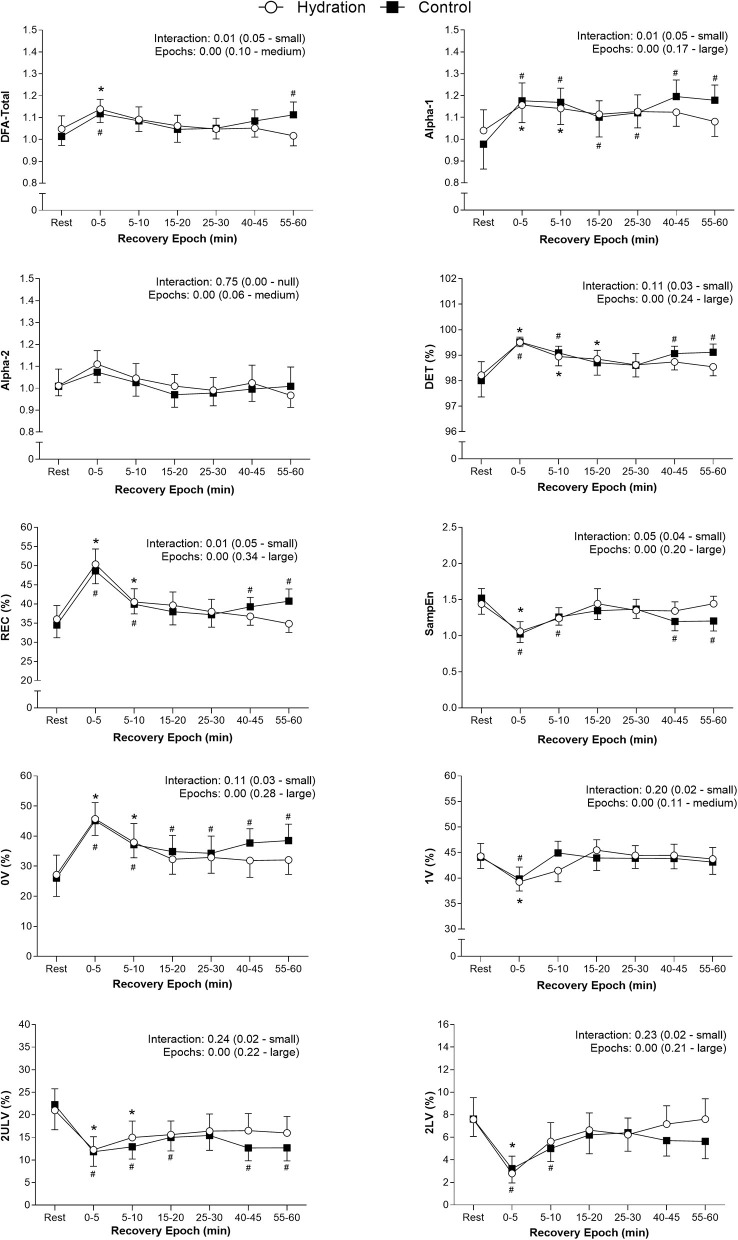
Differences between rest and recovery epochs for all indices analyzed in both protocols. Values are plotted as mean and 95% confidence intervals. The ANOVA effects found for epochs and interaction are presented as the p-value (effect size—effect magnitude). ^#^Significant difference (*p* < 0.05) from rest in CON; *Significant difference (*p* < 0.05) from rest in HYD; REC, Recurrence rate; DET, Determinism; SampEn, Sample entropy; DFA, detrended fluctuations analysis; 0V, no variation; 1V, one variation; 2LV, two equal variations; 2ULV, two different variations.

The non-linear HRV indices analyzed during recovery are shown in Figure 3. For all indices, there were no significant differences between protocols. However, significant time vs. protocol interaction was found for REC, DFA-total, and Alpha-1 indices (*P*-value < 0.05). There were significant differences between epochs (*P*-value < 0.05) for all indices.

Figure 3 shows that, during recovery, a gradual re-establishment of the indices to values close to those recorded at rest was observed in both protocols. There was a progressive increase of SampEn, 1V, 2LV, and 2ULV indices and a decrease of REC, DET, Alpha-1, Alpha-2, DFA-total, and 0V indices. However, the epoch in which this re-establishment occurred was different between protocols, happening earlier in HYD for most indices. Only the 1V index recovered at the same time in both protocols.

Table 4 displays results for SBP, DBP, and MBP. During exercise, there was no difference between protocols (SBP: *p* = 0.94; DBP: 0.91; MBP: 0.92) and no time vs. protocol interaction (SBP: *p* = 0.24; DBP: 0.47; MBP: 0.37). However, a large effect size was observed for SBP (*p* = 0.00; η^2^P = 0.62), DBP (*p* = 0.00; η^2^P = 0.24), and MBP (*p* = 0.00; η^2^P = 0.45). Similarly, analysis of the recovery showed no difference between protocols (SBP: *p* = 0.37; DBP: *p* = 0.56; MBP: 0.45) and no interaction (SBP: *p* = 0.63; DBP: *p* = 0.67; MBP: 0.63); however, a medium/large time effect was observed (SBP: *p* = 0.00; η^2^P = 0.08/DBP: *p* = 0.01; η^2^P = 0.05/MBP: 0.00; η^2^P = 0.47).

**Table 4:** Values of systolic, diastolic, and mean arterial blood pressure throughout control and hydration protocols (*N* = 30).

	**Rest**	**Exercise**		**Recovery**						
	**10°min**	**15°min**	**35°min**	**5°min**	**10°min**	**20°min**	**30°min**	**40°min**	**50°min**	**60°min**
**SBP (mmHg)**										
Control	116.3 ± 8.9	**132.1** **±12.0**	**132.1** **±11.1**	114.3 ± 10.1	112.3 ± 9.3	**110.0** **±9.8**	**108.3** **±9.8**	**109.3** **±9.8**	**111.3** **±14.8**	112.3 ± 13.8
	**–**	**–**	**–**	[−2.0 ± 9.6]	[−4.0 ± 8.1]	[−6.3 ± 9.3]	[−8.0 ± 9.6]	[−7.0 ± 9.1]	[−5.0 ± 14.6]	[−4.0 ± 13.8]
Hydration	118.0 ± 11.6	**132.3** **±9.3**	**129.7** **±10.3**	114.3 ± 13.8	113.3 ± 12.4	112.3 ± 12.5	112.3 ± 11.0	113.7 ± 14.0	114.3 ± 14.5	114.7 ± 14.3
	**–**	**–**	**–**	[−3.7 ± 11.6]	[−4.7 ± 10.7]	[−5.7 ± 11.6]	[−5.7 ± 12.2]	[−4.3 ± 11.9]	[−3.7 ± 11.9]	[−3.3 ± 12.9]
**DBP (mmHg)**										
Control	76.0 ± 7.7	**83.0** **±6.5**	79.0 ± 7.1	74.3 ± 8.2	71.7 ± 10.2	71.3 ± 9.4	71.3 ± 8.6	72.3 ± 9.7	72.7 ± 11.1	73.3 ± 12.1
	**–**	**–**	**–**	[−1.7 ± 7.5]	[−4.3 ± 8.6]	[−4.7 ± 8.6]	[−4.7 ± 7.7]	[−3.7 ± 8.1]	[3.3 ± 10.6]	[−2.7 ± 11.1]
Hydration	77.0 ± 11.5	**81.7** **±10.8**	78.7 ± 9.4	74.0 ± 10.0	73.0 ± 9.5	74.0 ± 10.4	74.0 ± 9.3	74.3 ± 10.1	73.0 ± 9.5	73.7 ± 9.6
	**–**	**–**	**–**	[−3.0 ± 7.9]	[−4.0 ± 8.9]	[−3.0 ± 9.9]	[−3.0 ± 11.8]	[−2.7 ± 9.8]	[−4.0 ± 10.4]	[−3.3 ± 9.9]
**MBP (mmHg)**										
Control	89.4 ± 7.5	**99.4** **±7.2**	**96.7** **±7.3**	87.7 ± 8.6	85.2 ± 9.5	**84.2** **±9.1**	**83.7** **±8.5**	**84.7** **±9.3**	**85.5** **±11.7**	86.3 ± 12.3
	**–**	–	–	[−1.8 ± 7.7]	[−4.2 ± 7.7]	[−5.2 ± 7.3]	[−5.8 ± 7.5]	[−4.8 ± 7.8]	[−3.9 ± 11.2]	[−3.1 ± 11.4]
Hydration	90.7 ± 10.5	**98.6** **±9.3**	**95.7** **±9.0**	87.4 ± 9.5	86.4 ± 9.0	86.8 ± 9.9	86.8 ± 8.9	87.4 ± 10.3	86.8 ± 10.6	87.3 ± 10.1
	**–**	**–**	**–**	[−3.2 ± 7.3]	[−4.2 ± 7.9]	[−3.9 ± 8.7]	[−3.9 ± 10.8]	[−3.2 ± 9.2]	[−3.9 ± 10.1]	[−3.3 ± 9.5]

Mean ± standard deviation [ΔRecovery–Rest]. Values in bold: significant difference (p < 0.05) from rest. SBP, systolic blood pressure; DBP, diastolic blood pressure; MBP, mean arterial blood pressure; mmHg, millimeters of mercury.

## 4. Discussion

Our main results showed that the water intake strategy could accelerate the recovery of the non-linear dynamic of HR of CAD subjects after an exercise-based cardiac rehabilitation session. However, during the exercise phase, the fluid lost and replaced did not influence the responses of the non-linear dynamics of HR in this period, corroborating with the previous studies of Silva et al. and Laurino et al.

The exercise protocol used in this study is similar to those commonly adopted in cardiac rehabilitation programs. The 0.7% reduction in body mass at the end of the CON showed that, although no significant changes were observed in the urine-specific gravity, the fluid loss registered approached a state of mild hypohydration, defined as a reduction of more than 1% in body mass (Mcdermott et al., 2017). It did not promote changes in the non-linear dynamics of HR during exercise, but it was able to delay the recovery of CAD subjects after a cardiac rehabilitation session.

The ANS is important in the regulation of acute hemodynamic responses to exercise. Parasympathetic withdrawal increases the HR in low-intensity exercise, and the activation of the sympathetic nervous system increases the HR at a level that will supply the body's demands in high-intensity exercise (Fisher et al., 2015). This scenario was observed in our study, as the non-linear autonomic responses observed were characterized by increased sympathetic and reduced parasympathetic modulation (evidenced by an increased 0V and a reduced 2LV and 2ULV, respectively). Also, a decreased complexity and fractal characteristic of the RR series is expected during exercise (evidenced by the decreased SampEn and increased REC, DET, DFA-total, and Alpha-2) (Casties et al., 2005; Floras and Ponikowski, 2015; Gronwald et al., 2018).

The same non-linear autonomic changes were observed in athletes during light and moderate aerobic exercise (Gronwald et al., 2018). These changes are explained by the sympathetic branch predominance, which reduces the variation between consecutive RR intervals, increasing the linearity and predictability and reducing the complexity of the series (Fisher et al., 2015; Gronwald et al., 2018).

The magnitude of autonomic responses to exercise is mediated by a complex pathway that includes central command, exercise pressor response, and arterial baroreflex modifications, all of which are associated with exercise intensity and environmental conditions (Fisher et al., 2015). The sympathetic overactivity observed in CAD (Ushijima et al., 2009) is related to chronotropic incompetence, fatigue, and diminished coronary blood flow during exercise (Fisher et al., 2015), augmenting the risk of chest pain and arrhythmias (Thompson et al., 2007). However, given the non-significant differences between the CON and HYD protocols, we can conclude that the water drinking was not able to change or enhance the autonomic profile of CAD during exercise by maintaining the hydration status.

It is important to highlight that the negative effects of fluid loss during exercise in heart rate, cardiac output, and arterial blood pressure are usually registered above ~2% of body mass reduction induced by higher exercise intensities and hot environments (Trangmar and González-Alonso, 2019), which were not part of our study. Also, the fluid loss occurred throughout the periods of exercise and recovery, so the amount of fluid lost only during exercise may not be sufficient to influence non-linear responses during this period.

Immediately after exercise, the HR rapidly decreases, mainly due to parasympathetic reactivation, mediated by the interruption of central command and the mechanoreflex (evidenced by the increase in 2LV and 2ULV). After this fast reduction, the HR remains slightly elevated due to the slow sympathetic activity reduction in the face of the gradual reestablishment of body temperature and reduction of metaboreflexes (evidenced by the decrease in 0V) (Peçanha et al., 2014). This residual sympathetic overactivity is important to maintain a slightly elevated cardiac output to preserve the perfusion pressure against peripheral vasodilatation (Fisher et al., 2015). During recovery, the complexity and fractal characteristics of the RR series are reestablished (evidenced by the increase in SampEn, the decrease in REC and DET, and the return of DFA-total and Alpha-1 to values closer to 1.00) (Godoy, 2016).

The ANS recovery in the CON protocol was delayed, and some indices did not achieve complete recovery after the 60-min evaluation. This delay, especially for CAD individuals, represents an increased risk, once the maintenance of a high sympathetic modulation induces ischemic and arrhythmic events during recovery (Thompson et al., 2007). Also, in the CON protocol, the REC, DET, SampEn, DFA-total, and 2ULV indices reached the recovery between the 10th and 30th min post-exercise. However, after 40 min, they returned to an unrecovered state, suggesting that maintaining a state of hypohydration after exercise may retard the long-term recovery of the ANS, which should be further investigated in future studies. However, when the water drinking strategy was used, all indices achieved complete recovery between the 5th and 20th min. These results are in accordance with previous studies (Laurino et al., 2021; Silva et al., 2022), reinforcing the positive effects of water drinking in the post-exercise autonomic recovery of CAD individuals.

The beneficial effects of water drinking on autonomic recovery in CAD are yet to be fully elucidated. However, based on previous observations, we may hypothesize that the sympathetic-mediated acute pressor response may be involved in this response. It is known that, at the level of portal circulation, small changes in blood osmolality toward hypoosmolality produce a spinal reflex-like mechanism that activates the postganglionic sympathetic neurons, leading to a rise in blood pressure. This pressor response was observed only in populations with an altered baroreflex sensitivity, as found in the elderly (May and Jordan, 2011), the population of our study. The results found for arterial blood pressure confirm this hypothesis; once in the HYD protocol, the post-exercise hypotension observed in the CON protocol was attenuated.

Although, due to the complexity of the neuronal pathways regulating cardiovascular function, a rise in sympathetic activity at the spinal level does not produce cardiac responses (May and Jordan, 2011). Our data confirm this mechanism, despite the high blood pressure during the recovery of the non-linear response, which indicated a more preserved parasympathetic modulation during the HYD protocol. In line with this theory, a recent study showed that the acute ingestion of at least 200 mL of water produced a bradycardia response related to an increased vagal tone at the sinus node. However, the physiological mechanism responsible for this response is not wellestablished in the literature (Grasser, 2020). It is important to point out that all these mechanisms were previously investigated in a rest condition in response to a single bout of water ingestion; thus, the post-exercise response to a fractioned dose drinking strategy may include different pathways.

The fluid replacement protocol proposed in this study is classified as “programmed or planned drinking,” in which a predetermined and individualized amount of fluid is ingested to prevent dehydration by matching the sweat losses. This method considers the variation in the sweating rate between individuals (Kenefick, 2018). Also, most of the individuals enrolled in cardiac rehabilitation are elderly and may have an impaired sense of thirst (Kenney and Chiu, 2001), which could preclude adequate fluid replacement if it is performed *ad libitum*, which is the method commonly used when the patient brings his water bottle to the rehabilitation session and drinks it during or at the end of the exercise.

In the clinical practice scenario, these results have important implications, as they raise the potential importance of the implementation of planned drinking protocols during exercise-based cardiac rehabilitation sessions. Also, this strategy is low-cost and easy to implement and could increase the safety of these programs (Thompson et al., 2007; Qiu et al., 2017). Also, we highlight the importance of controlling the temperature of the cardiac rehabilitation room and orienting the patients regarding the importance of fluid replacement and the place and time of day to perform unsupervised activities. This is because high temperatures can promote even more significant fluid losses that, when not adequately replaced, may significantly impair autonomic recovery, increasing the risks of sudden events. Furthermore, this study adds new information to the literature regarding the characteristics and physiology of the non-linear dynamics of HR of CAD subjects during the periods of exercise and recovery. To our knowledge, this is the first study to address it and consider the effects of fluid loss as well as its replacement in these periods.

Despite these promising results, the limitation regarding the lack of randomization needs to be pointed out. Thus, to minimize this potential bias, only individuals familiar with and attending cardiac rehabilitation were included to eliminate the possible acute effects of exercise.

New studies addressing this topic are needed to better understand the physiological effects of fluid loss and replacement in subjects with CAD, the effects of using other fluid replacement strategies, and the use of other types of beverages. The effect of water drinking when active recovery is performed needs to be investigated. As we included only males in our study, new research on the female population is needed as well.

Given these results, we conclude that the proposed water drinking protocol was able to accelerate the recovery of non-linear dynamics of HR after moderate-intensity aerobic exercise performed in the models of a cardiac rehabilitation session and that the volume of fluid lost during exercise did not influence autonomic responses in this period.

## Data availability statement

The raw data supporting the conclusions of this article will be made available by the authors, without undue reservation.

## Ethics statement

The studies involving human participants were reviewed and approved by Committee for Ethics and Research of the Faculty of Science and Technology—UNESP (CAAE: 54864716.8.0000.5402). The patients/participants provided their written informed consent to participate in this study.

## Author contributions

AS and LV conceived and designed the study. ML, AS, and LS carried out the experiment and data collection. ML and AS performed the data analysis. ML, AS, and LV contributed to the data interpretation and manuscript writing. LV supervised the project. All authors discussed and contributed to the final manuscript version.

## References

[B1] American College of Sports Medicine. (2014). ACSM‘s Guidelines for Exercise Testing and Prescription. 9 ed. Baltimore: Wolters Kluwer. 10.1249/JSR.0b013e31829a68cf 23851406

[B2] Armstrong L. E. (2005). Hydration assessment techniques. Nutr. Rev. 63, S40–S54. 10.1301/nr.2005.jun.S40-S54 16028571

[B3] Cabral-Santos C. Gerosa-Neto J. Inoue D. S. Panissa V. L. G. Gobbo L. A. Zagatto A. M.. (2015). Similar anti-inflammatory acute responses from moderate-intensity continuous and high-intensity intermittent exercise. J. Sport Sci. Med. 14, 849–856. 26664283 PMC4657429

[B4] Casties J. Mottet D. Le Gallais D. (2005). Non-Linear analyses of heart rate variability during heavy exercise and recovery in cyclists. Int. J. Sports Med. 26, 1–6. 10.1055/s-2005-872968 16586334

[B5] Catai A. M. Pastre C. M. Godoy M. F. de Silva E. Takahashi A. C. de Siva M.. (2019). Heart rate variability: are you using it properly? Standardization checklist of procedures. Braz. J. Phys. Ther. 20, 215. 10.1016/j.bjpt.2019.02.006 30852243 PMC7082649

[B6] Dabiré H. Mestivier D. Jarnet J. Safar M. E. Chau N. P. (1998). Quantification of sympathetic and parasympathetic tones by non-linear indexes in normotensive rats. Am. J. Physiol. Heart. Circ. Physiol. 275, 1290–1297. 10.1152/ajpheart.1998.275.4.H1290 9746478

[B7] Dwan K. Li T. Altman D. G. Elbourne D. (2019). CONSORT 2010 statement: extension to randomized crossover trials. Bmj 366, l4378. 10.1136/bmj.l4378 31366597 PMC6667942

[B8] Eckmann J. P. Kamphorst S. O. Ruelle D. (1987). Recurrence plots of dynamical systems. Europhys. Lett. 4, 973–977. 10.1209/0295-5075/4/9/004

[B9] Figueiredo R. Pereira R. Neto O. P. (2018). Non-linear analysis is the most suitable method to detect changes in heart autonomic control after exercise of different durations. Comput. Biol. Med. 97, 83–88. 10.1016/j.compbiomed.2018.04.011 29709717

[B10] Fisher J. P. Young C. N. Fadel P. J. (2015). Autonomic adjustments to exercise in humans. Compr. Physiol. 5, 475–512. 10.1002/cphy.c140022 25880502

[B11] Floras J. S. Ponikowski P. (2015). The sympathetic/parasympathetic imbalance in heart failure with reduced ejection fraction. Eur. Heart J. 36, 1974–1982. 10.1093/eurheartj/ehv087 25975657 PMC4528097

[B12] Godoy M. F. (2016). Non-linear analysis of heart rate variability: a comprehensive review. J. Cardiol. Therapy 3, 528–533. 10.17554/j.issn.2309-6861.2016.03.101-4

[B13] Goldberger A. L. Amaral L. A. Glass L. Hausdorff J. M. Ivanov P. C. Mark R. G. (2000). PhysioBank, PhysioToolkit and PhysioNet: Components of a new research resource for complex physiologic signals. Circulation. 101, E215–E220. 10.1161/01.CIR.101.23.e215 10851218

[B14] Grasser E. K. (2020). Dose-dependent heart rate responses to drinking water: a randomized crossover study in young, non-obese males. Clinic. Autonom. Res. 30, 567–570. 10.1007/s10286-020-00673-6 32078090

[B15] Gronwald T. Hoos O. Ludyga S. Hottenrott K. (2018). Non-linear dynamics of heart rate variability during incremental cycling exercise. Res. Sports Med. 8, 1–11. 10.1080/15438627.2018.1502182 30040499

[B16] Harvey R. Dolgin R. Leom T. (1994). “New York Heart Association Criteria Committe,” in Nomenclature and Criteria for Diagnosis of Diseases of the Heart and Great Vessels (Boston: Little. Brown). p. 286.

[B17] Henriques T. Ribeiro M. Teixeira A. Castro L. Antunes L. Costa-Santos C. (2020). Non-linear methods most applied to heart-rate time series: a review. Entropy 22, 309. 10.3390/e22030309 33286083 PMC7516766

[B18] Javorka M. Zila I. Balharek T. Javorka K. (2002). Heart rate recovery after exercise: relations to heart rate variability and complexity. Brazil. J. Med. Biologic. Res. 35, 991–1000. 10.1590/S0100-879X2002000800018 12185393

[B19] Kenefick R. W. (2018). Drinking strategies: planned drinking vs. drinking to thirst. Sports Med. 48, 31–37. 10.1007/s40279-017-0844-6 29368181 PMC5790864

[B20] Kenney W. L. Chiu P. (2001). Influence of age on thirst and fluid intake. Med. Sci. Sports Exerc. 33, 1524–1532. 10.1097/00005768-200109000-00016 11528342

[B21] Laurino M. J. L. da Silva A. K. F. Santos L. A. Ribeiro F. Vanzella L. M. Corazza D. A. G.. (2021). Vagal reactivation after a cardiac rehabilitation session associated with hydration in coronary artery disease patients: crossover clinical trial. Sci. Rep. 11, 40. 10.1038/s41598-021-89840-x 34006912 PMC8131702

[B22] Lima C. T. Freire A. C. Silva A. P. Teixeira R. M. Farrell M. Prince M. (2005). Concurrent and construct validity of the audit in an urban brazilian sample. Alcohol Alcohol 40, 584–589. 10.1093/alcalc/agh202 16143704

[B23] Maher J. M. Markey J. C. Ebert-May D. (2013). The other half of the story: Effect size analysis in quantitative research. CBE Life Sci. Educ. 12, 345–351. 10.1187/cbe.13-04-0082 24006382 PMC3763001

[B24] May M. Jordan J. (2011). The osmopressor response to water drinking. Am. J. Physiol. Regul. Integr. Comp. Physiol. 300, 40–46. 10.1152/ajpregu.00544.2010 21048076

[B25] Mcdermott B. P. Anderson S. A. Lawrence E. Casa D. J. Samuel N. Cooper L.. (2017). National athletic trainers' association position statement: fluid replacement for the physically active. J. Athl. Train. 52, 877–895. 10.4085/1062-6050-52.9.02 28985128 PMC5634236

[B26] Meneghelo R. S. Araújo C. G. S. Stein R. Mastrocolla L. E. Albuquerque P. F. Serra S. M. (2011). III Diretrizes da Sociedade Brasileira de Cardiologia Sobre Teste Ergométrico. Rev. Bras. Fisioter. 95, 1–26. 10.1590/S0066-782X201000240000121340292

[B27] Nunan D. Gay D. Jakovljevic D. G. Hodges L. D. Sandercock G. R. H. Brodie D. A. (2009). Validity and reliability of short-term heart-rate variability from the Polar S810. Med. Sci. Sports Exer. 41, 243–250. 10.1249/MSS.0b013e318184a4b1 19092682

[B28] Peçanha T. Bartels R. Brito L. C. Paula-Ribeiro M. Oliveira R. S. Goldberger J. J. (2017). Methods of assessment of the post-exercise cardiac autonomic recovery: a methodological review. Int. J. Cardiol. 227, 795–802. 10.1016/j.ijcard.2016.10.057 27836300

[B29] Peçanha T. Silva-Júnior N. D. Forjaz C. L. de M. (2014). Heart rate recovery: autonomic determinants, methods of assessment and association with mortality and cardiovascular diseases. Clin. Physiol. Funct. Imag. 34, 327–339. 10.1111/cpf.12102 24237859

[B30] Peng C. K. Havlin S. Stanley H. E. Goldberger A. L. (1994). Quantification of scaling exponents and crossover phenomena in non-stationary heartbeat time series. CHAOS 5, 82–87. 10.1063/1.166141 11538314

[B31] Porta A. Rienzo M. di Wessel, N. Kurths J. (2009). Addressing the complexity of cardiovascular regulation. Philosophic. Transact. Royal Soc. Mathematic. Physic. Eng. Sci. 367, 1215–1218. 10.1098/rsta.2008.0292 19324704

[B32] Porta A. Tobaldini E. Guzzetti S. Furlan R. Montano N. Gnecchi-ruscone T. (2007). Assessment of cardiac autonomic modulation during graded head-up tilt by symbolic analysis of heart rate variability. Am. J. Physiol. Heart Circ. Physiol. 293, 702–708. 10.1152/ajpheart.00006.2007 17308016

[B33] Qiu S. Cai X. Sun Z. Li L. Zuegel M. Steinacker J. M.. (2017). Heart rate recovery and risk of cardiovascular events and all-cause. J. Am. Heart Assoc. 6, 1–12. 10.1161/JAHA.117.005505 28487388 PMC5524096

[B34] Rezende Barbosa M. P. C. Silva N. T. Azevedo F. M. Pastre C. M. Vanderlei L. C. M. (2016). Comparison of Polar^®^ RS800G3TM heart rate monitor with Polar^®^ S810iTM and electrocardiogram to obtain the series of RR intervals and analysis of heart rate variability at rest. Clin. Physiol. Funct. Imag. 36, 112–117. 10.1111/cpf.12203 25348547

[B35] Richman J. S. Moorman J. R. (2000). Physiological time-series analysis using approximate entropy and sample entropy. Am. J. Physiol. Heart Circ. Physiol. 278, 2039–2049. 10.1152/ajpheart.2000.278.6.H2039 10843903

[B36] Sawka M. N. Burke L. M. Eichner E. R. Maughan R. J. Montain S. J. Stachenfeld N. S. (2007). Exercise and fluid replacement. Med. Sci. Sport Exer. 39, 377–390. 10.1249/mss.0b013e31802ca597 17277604

[B37] Shaffer F. Ginsberg J. P. (2017). An Overview of heart rate variability metrics and norms. Front. Public Health 5, 258. 10.3389/fpubh.2017.00258 29034226 PMC5624990

[B38] Silva A. K. F. Laurino M. J. L. Vanzella L. M. Santos L. A. Ribeiro F. Corazza D. A. G.. (2020). Influence of the hydration on autonomic modulation and cardiorespiratory parameters of coronary heart disease patients submitted to a cardiovascular rehabilitation session: Crossover clinical trial protocol. Motriz. Revista de Educacao Fisica 26, 2210. 10.1590/s1980-6574202000010022

[B39] Silva A. K. F. Santos L. A. Laurino M. J. L. Vanzella L. M. Ribeiro F. Rozan G. B.. (2022). Hydration influence on the autonomic recovery of the coronary diseases patient: geometric indices analysis. Res. Q. Exerc. Sport 93, 230–239. 10.1080/02701367.2020.1818672 32976086

[B40] Tarvainen M. P. Niskanen J. P. Lipponen J. A. Ranta-aho P. O. Karjalainen P. A. (2014). Kubios HRV—heart rate variability analysis software. Comput. Meth. Prog. Bio. 113, 210–220. 10.1016/j.cmpb.2013.07.024 24054542

[B41] Thompson P. D. Franklin B. A. Balady G. J. Blair S. N. Corrado D. Estes N. A. M.. (2007). Exercise and acute cardiovascular events: Placing the risks into perspective a scientific statement from the american heart association council on nutrition, physical activity, and metabolism and the council on clinical cardiology. Circulation 115, 2358–2368. 10.1161/CIRCULATIONAHA.107.181485 17468391

[B42] Trangmar S. J. González-Alonso J. (2019). Heat, hydration and the human brain, heart and skeletal muscles. Sports Med. 49, S69–S85. 10.1007/s40279-018-1033-y 30671905 PMC6445826

[B43] Ushijima A. Fukuma N. Kato Y. Aisu N. Mizuno K. (2009). Sympathetic excitation during exercise as a cause of attenuated heart rate recovery in patients with myocardial infarction. J. Nippon Med. Sch. 76, 76–83. 10.1272/jnms.76.76 19443992

[B44] Von Duvillard S. P. Braun W. A. Markofski M. Beneke R. Leithäuser R. (2004). Fluids and hydration in prolonged endurance performance. Nutrition 20, 651–656. 10.1016/j.nut.2004.04.011 15212747

